# Acute and Subacute Oral Toxicity Evaluation of *Baccaurea motleyana* Fruit Extract in Wistar Rats

**DOI:** 10.1002/fsn3.71744

**Published:** 2026-04-09

**Authors:** Md. Mahmudul Hasan, Eftekhar Alam Bhuyan, Md. Ekramul Haque Ekram, Nawreen Monir Proma, Mohammed Kamrul Hossain

**Affiliations:** ^1^ Department of Pharmacy University of Chittagong Chittagong Bangladesh; ^2^ Department of Dentistry Chittagong Medical University Chittagong Bangladesh

**Keywords:** *Baccaurea motleyana*, *hematology*, *histopathology*, *organ toxicity*, *toxicity*

## Abstract

*Baccaurea motleyana* (Rambai) is traditionally consumed in Southeast Asia and used ethnomedicinally, yet its toxicological profile remains unclear. This study evaluated the acute and subacute oral toxicity of its aqueous fruit extract (AE‐BMF) in Wistar rats. AE‐BMF was prepared from fresh pulp. Acute toxicity was assessed at 2000 mg/kg, while subacute toxicity was tested for 28 days at 400 mg/kg/day and 800 mg/kg/day. Physiological, hematological, biochemical, and histopathological parameters were evaluated. No mortality or overt toxicity was observed in the acute study (LD_50_ > 2000 mg/kg). In the subacute study, AE‐BMF at 800 mg/kg reduced body weight gain, water intake, and food intake, and increased relative liver weight by 15.8% (*p* < 0.05). Biochemical analysis showed reductions in creatinine (0.50 ± 0.03 mg/dL, *p* < 0.01), uric acid (9.1 ± 0.6 mg/dL, *p* < 0.01), total cholesterol (73.5 ± 3.1 mg/dL, *p* < 0.01), and triglycerides (39.8 ± 3.3 mg/dL, *p* < 0.001) at 800 mg/kg dose. ALT rose moderately at 800 mg/kg (108.4 ± 6.0 U/L, *p* < 0.05). Hematological changes included increased RBC (7.70 ± 0.28 × 10^6^/mm^3^, *p* < 0.01), hemoglobin (14.4 ± 0.3 g/dL, *p* < 0.001), platelets (585 ± 18 × 10^3^/mm^3^, *p* < 0.001), and lymphocytes (57.3% ± 1.9%, *p* < 0.01). Histology confirmed normal kidneys with only mild hepatic alterations at a dose of 800 mg/kg. AE‐BMF is safe at doses up to 800 mg/kg, demonstrating hypolipidemic, hematopoietic, and immunostimulatory effects. Mild hepatic changes at higher doses suggest the need for cautious use and longer‐term studies.

## Graphical abstract

*Baccaurea motleyana* fruit exhibited no significant toxic effects in Wistar rats, as evidenced by hematological and biochemical analyses. Moreover, histopathological evaluation of the liver and kidney revealed no detectable abnormalities.

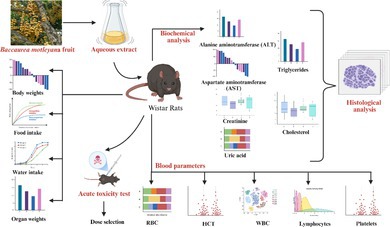

**Abbreviations**
AE‐BMF: aqueous extract of *Baccaurea motleyana* fruit
ALT: alanine aminotransferase
AST: aspartate aminotransferase
H&E: hematoxylin and eosin
Hb: hemoglobin
HCT: hematocrit
LD_50_
: median lethal dose
OECD: organization for economic co‐operation and development
RBC: red blood cell
SEM: standard error of the mean
WBC: white blood cell

## 1 Introduction

Plants have long served as vital sources of therapeutic agents, with a rich history tracing back thousands of years. Currently, a significant proportion of the global population, especially in developing regions, continues to rely primarily on plant‐based traditional healthcare (Hasan, Wasin, et al. 2025). Despite the existence of hundreds of thousands of plant secondary metabolites, only a fraction have been extensively explored for medicinal use (Hasan, Alim, et al. 2025). This underscores the potential of underutilized plants as promising candidates for novel drug discovery.

*Baccaurea motleyana* Müll. Arg. (generally known as Rambai), falls under the *Phyllanthaceae* family and is native to Southeast Asia, including Sumatra, Peninsular Malaysia, Java, Borneo, Thailand, and parts of Bangladesh and India (Prodhan and Mridu 2023). The tree can reach heights of 9 m to 25 m and produces small, globose to slightly flattened fruits with translucent, white pulp that tastes sweet to sour. Rambai is still underexplored therapeutically despite its nutritional and traditional usage (Debnath et al. 2022a). Recently, *B. motleyana* has been reported to be a rich source of vitamins, dietary fiber, minerals, and diverse phytochemicals, including phenolic acids, flavonoids, carotenoids, terpenes, and organic acids such as citric, tartaric, and malic (Debnath et al. 2022a); (Riti et al. 2025a). Its pulp exhibits moderate antioxidant activity, primarily attributed to its ascorbic acid and β‐carotene content (Riti et al. 2025b) In traditional practices, different parts of the plant are used ethnomedicinally. Fresh or processed fruit is consumed in juice, organic vinegar, jams, and wine (Debnath et al. 2022a). In treating skin disorders, postpartum care, eye inflammation, and women's health issues such as menstrual disorders, the bark has noteworthy applications (Sany et al. 2025); (Shompa et al. 2024), while also being used to treat skin conditions and abdominal pain (Fransiska et al. 2024), (Shompa et al. 2024). To substantiate the therapeutic potential of *B. motleyana*, scientific investigations have been initiated. Significant hypoglycemic, antidepressant, antidiarrheal, and moderate antimicrobial effects in animal models were imparted by a methanolic seed extract (Shompa et al. 2024). The presence of flavonoids, alkaloids, tannins, saponins, glycosides, and steroids/triterpenoids was further confirmed by the phytochemical screenings of leaf and bark extracts (Sany et al. 2025).

Despite the common perception that medical plants and edible botanicals are safe, growing evidence indicates that phytomedicines may cause adverse effects, particularly at elevated doses, with prolonged use, or in the absence of appropriate standards (Teschke and Eickhoff 2015). Epidemiological investigations have indicated hepatotoxicity, nephrotoxicity, and herb–drug interactions associated with traditional herbal products (Herb–drug interactions: an overview of systematic reviews). *B. motleyana* is widely used for its fruit and in traditional medicine in Southeast Asia (Hasan, Azme, et al. 2025). The liver and kidney were selected because they are primary targets for the metabolism and excretion of xenobiotics, making them more likely to exhibit early signs of toxicity (Alnasser 2025). Nonetheless, despite its abundance of phytochemical constituents and growing pharmacological evidence, its toxicity profile remains inadequately defined. The disparity between extensive human exposure and the absence of preclinical safety evidence indicates a critical knowledge gap and a potential public health concern. This study aims to analyze the acute and subacute oral toxicity of the aqueous extract of *B. motleyana* fruit (AE‐BMF) in Wistar rats by evaluating dose‐dependent physiological, biochemical, hematological, and histopathological parameters to determine its preliminary safety profile.

## 2 Materials and Methods

### 2.1 Plant Material and Preparation of Aqueous Extract

From the local farm in Narsingdi, Bangladesh, fresh ripe fruits of *B. motleyana* were collected. A taxonomist at the Department of Botany, University of Chittagong, where a voucher specimen (MMH‐CUDP‐06‐24) was deposited, authenticated the fruits. Using a blender, the fruit pulp was separated, washed thoroughly, and homogenized. At room temperature for 24 h with intermittent stirring, the homogenate was macerated in distilled water (1:5 w/v). Filtration of the mixture was conducted first through muslin cloth, then through Whatman No. 1 filter paper. The filtrate was then frozen at −20°C and subsequently lyophilized using a freeze‐dryer (Labconco, USA) to yield a dry powder. At 4°C in airtight containers, the lyophilized aqueous extract of *B. motleyana* fruit (AE‐BMF) was stored until use. For administration, AE‐BMF was freshly dissolved in distilled water (Mitsagga et al. 2021).

### 2.2 Experimental Animals

Healthy Wistar albino rats of both sexes, aged 6 to 8 weeks and weighing 120 g to 150 g, were obtained from ICDDRb, Chattogram. Animals were maintained in polypropylene cages under regulated environmental parameters (temperature, 25°C ± 2°C; 12 h light/dark cycle; relative humidity, 50%–60%). A standard pellet diet and water were provided. Rats underwent a two‐week acclimatization period before experimentation. All experimental procedures were conducted in accordance with the guidelines established by the Ethical Committee of the University of Chittagong, under ethical approval number AERB‐FBSCU‐20250615‐(1).

### 2.3 Acute Oral Toxicity Study

An acute oral toxicity study was conducted in rats in accordance with OECD Guideline 425 (Up‐and‐Down Procedure) (Sewell et al. 2024). A limit dose of 2000 mg/kg of the extract was administered by oral gavage after a 3 h fast, as recommended for substances with low expected toxicity, such as edible plant products. The animals were observed closely for signs of toxicity during the first 24 h. Subsequently, four additional rats received the same dose and were monitored for 14 days for morbidity and mortality. No mortality or severe toxic signs were observed in any animal. Therefore, the median lethal dose (LD_50_) was considered to be greater than 2000 mg/kg. Based on this estimated LD_50_, the doses for the sub‐acute study were selected as fractions of the highest non‐lethal dose. Therapeutic doses were set at one‐fifth (400 mg/kg) and two‐fifths (800 mg/kg) of the anticipated LD_50_ for further subacute toxicity assessment.

### 2.4 Subacute Toxicity Study

The subacute study was performed in compliance with OECD Guideline 407 (Kunimatsu et al. 2004). A total of 30 rats, comprising 15 males and 15 females, were randomly assigned to three groups (*n* = 10, with five males and five females in each group):

– Group I: Vehicle control (distilled water, 10 mL/kg)
– Group II: AE‐BMF 400 mg/kg
– Group III: AE‐BMF 800 mg/kg

For 28 consecutive days, extracts were administered orally once daily. Body weight, water consumption, and food intake were documented weekly. Upon completion of the experimental period, the animals underwent an overnight fast, were anesthetized with ketamine (50 mg/kg) and diazepam (10 mg/kg), and were subsequently sacrificed. For hematological and biochemical analysis, blood was obtained through cardiac puncture. Vital organs (liver, kidneys, heart, lungs, spleen) were excised, blotted, and weighed for calculation of relative organ weight. In 10% neutral‐buffered formalin, samples of liver and kidney were fixed for histopathological examination (Nalimu et al. 2022).

### 2.5 Biochemical Analysis

At the conclusion of the experimental period, blood was collected from Wistar rats via cardiac puncture for biochemical evaluation. At room temperature, the samples were allowed to clot, and the serum was then separated by centrifugation at 3000 rpm for 15 min. The serum was analyzed using an automated biochemical analyzer according to the manufacturer's instructions, with commercially available diagnostic kits. The measured parameters comprised alanine aminotransferase (ALT), aspartate aminotransferase (AST), creatinine, total cholesterol, uric acid, and triglycerides (Yustisia et al. 2022).

### 2.6 Hematological Analysis

By assessing hematological parameters, the impact of AE‐BMF on blood profiles was evaluated. At the conclusion of the treatment period, blood samples were collected from Wistar rats via cardiac puncture, using EDTA‐coated tubes. To assess the red blood cell (RBC) count, white blood cell (WBC) count, lymphocyte count, hemoglobin (Hb) concentration, hematocrit (HCT), and platelet count, the samples were promptly analyzed with an automated hematology analyzer (Figueroa‐Mujica et al. 2022).

### 2.7 Histopathological Examination

In 10% buffered formalin, liver and kidney tissues were fixed, dehydrated using graded ethanol, cleared with xylene, and subsequently embedded in paraffin. Using a rotary microtome, sections measuring 5 μm were prepared and subsequently stained with hematoxylin and eosin (H&E) for further analysis. Using a light microscope (Olympus, Japan), slides were analyzed to identify any histological changes (Yao et al. 2021).

### 2.8 Statistical Analysis

Data were presented as mean ± SEM. A one‐way analysis of variance (ANOVA) was conducted, followed by Tukey's post hoc test for group comparisons. A *p*‐value of less than 0.05 was deemed statistically significant. Statistical analyses were performed using GraphPad Prism 8.0.

## 3 Results

### 3.1 Acute Toxicity Study

Oral administration of AE‐BMF at a single dose of 2000 mg/kg produced no observable signs of toxicity or mortality during the 14 day monitoring period. The median lethal dose (LD_50_) of AE‐BMF was therefore estimated to be greater than 2000 mg/kg.

### 3.2 Subacute Toxicity Study

**FIGURE 1:**
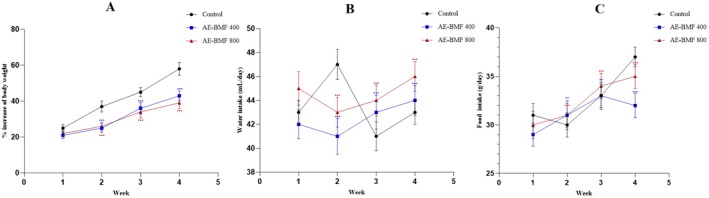
Changes of body weight (A), water intake (B), and food intake (C) after the treatment. Data were represented as mean ± SEM. Sample size *n* = 10. Significance at *p < 0.05, **p < 0.01, and ***p < 0.001. AE‐BMF, Aqueous extract of *B. motleyana* fruit.

During the 28 day treatment period, no mortality or marked behavioral alterations were observed in the treated groups. In the 4th week, AE‐BMF 800 mg/kg significantly reduced body weight gain, water intake & food consumption compared with controls (*p* < 0.05), whereas AE‐BMF 400 mg/kg showed only mild changes (Figure 1). No significant differences were observed in the relative weights of kidneys, lungs, spleen & pancreas at 400 mg/kg. A modest but significant increase in relative liver weight was noted in both male and female rats at AE‐BMF 800 mg/kg (Table 1).

**TABLE 1:** Relative organ weights of the rats after treatment.

Organ	Control	AE‐BMF 400 mg/kg	AE‐BMF 800 mg/kg
Liver	4.68 ± 0.22	4.83 ± 0.18	5.42 ± 0.20**
Kidneys	0.50 ± 0.02	0.51 ± 0.03	0.52 ± 0.02*
Heart	0.34 ± 0.01	0.36 ± 0.02	0.37 ± 0.01
Lungs	0.91 ± 0.03	0.88 ± 0.02	0.89 ± 0.04
Spleen	0.40 ± 0.02	0.41 ± 0.02	0.42 ± 0.03***

*Note:* Data were represented as mean ± SEM. Sample size *n* = 10.

Abbreviation: AE‐BMF, aqueous extract of *B. motleyana* fruit.

* *p* < 0.05.

** *p* < 0.01.

*** *p* < 0.001.

### 3.3 Biochemical Parameters

At 400 and 800 mg/kg, AE‐BMF significantly reduced creatinine, uric acid, total cholesterol, and triglyceride levels in a dose‐dependent manner, with greater effects at the higher dose. ALT increased slightly at 800 mg/kg, while AST remained essentially unchanged across groups (Table 2).

**TABLE 2:** Biochemical parameters of the rats after treatment.

Parameter	Control	AE‐BMF 400 mg/kg	AE‐BMF 800 mg/kg
ALT (U/L)	89.2 ± 5.1	92.6 ± 4.7	108.4 ± 6.0*
AST (U/L)	92.1 ± 6.3	88.4 ± 5.9	95.6 ± 7.2
Creatinine (mg/dL)	0.72 ± 0.05	0.64 ± 0.04	0.50 ± 0.03**
Uric acid (mg/dL)	12.8 ± 0.9	10.9 ± 0.7*	9.1 ± 0.6**
Total cholesterol (mg/dL)	101.4 ± 4.5	87.2 ± 3.8*	73.5 ± 3.1**
Triglycerides (mg/dL)	70.5 ± 4.2	52.4 ± 3.6**	39.8 ± 3.3***

*Note:* Data were represented as mean ± SEM. Sample size *n* = 10.

Abbreviation: AE‐BMF, aqueous extract of *B. motleyana* fruit.

* *p* < 0.05.

** *p* < 0.01.

*** *p* < 0.001.

### 3.4 Hematological Parameters

The hematological data indicate that AE‐BMF treatment enhances erythropoietic and immune parameters. Both 400 and 800 mg/kg doses significantly increased RBC and hemoglobin levels, with the effect being more pronounced at 800 mg/kg. Platelet counts rose markedly at both treatment doses, whereas lymphocyte percentage increased markedly, particularly at 800 mg/kg. Hematocrit and WBC counts remained relatively stable across groups (Table 3).

**TABLE 3:** Hematological parameters of the rats after treatment.

Parameter	Control	AE‐BMF 400 mg/kg	AE‐BMF 800 mg/kg
RBC (×10^6^/mm^3^)	7.40 ± 0.25	7.58 ± 0.32*	7.70 ± 0.28**
Hb (g/dL)	13.9 ± 0.4	14.2 ± 0.5**	14.4 ± 0.3***
HCT (%)	52.8 ± 1.1	53.4 ± 1.3	51.6 ± 1.2
WBC (×10^3^/mm^3^)	9.5 ± 0.4	9.8 ± 0.5	10.0 ± 0.6
Lymphocytes (%)	46.2 ± 1.4	47.5 ± 1.6	57.3 ± 1.9
Platelets (×10^3^/mm^3^)	505 ± 12	580 ± 14***	585 ± 18***

*Note:* Data were represented as mean ± SEM. Sample size *n* = 10.

Abbreviation: AE‐BMF, aqueous extract of *B. motleyana* fruit.

* *p* < 0.05.

** *p* < 0.01.

*** *p* < 0.001.

### 3.5 Histological Analysis of Liver and Kidney

**FIGURE 2:**
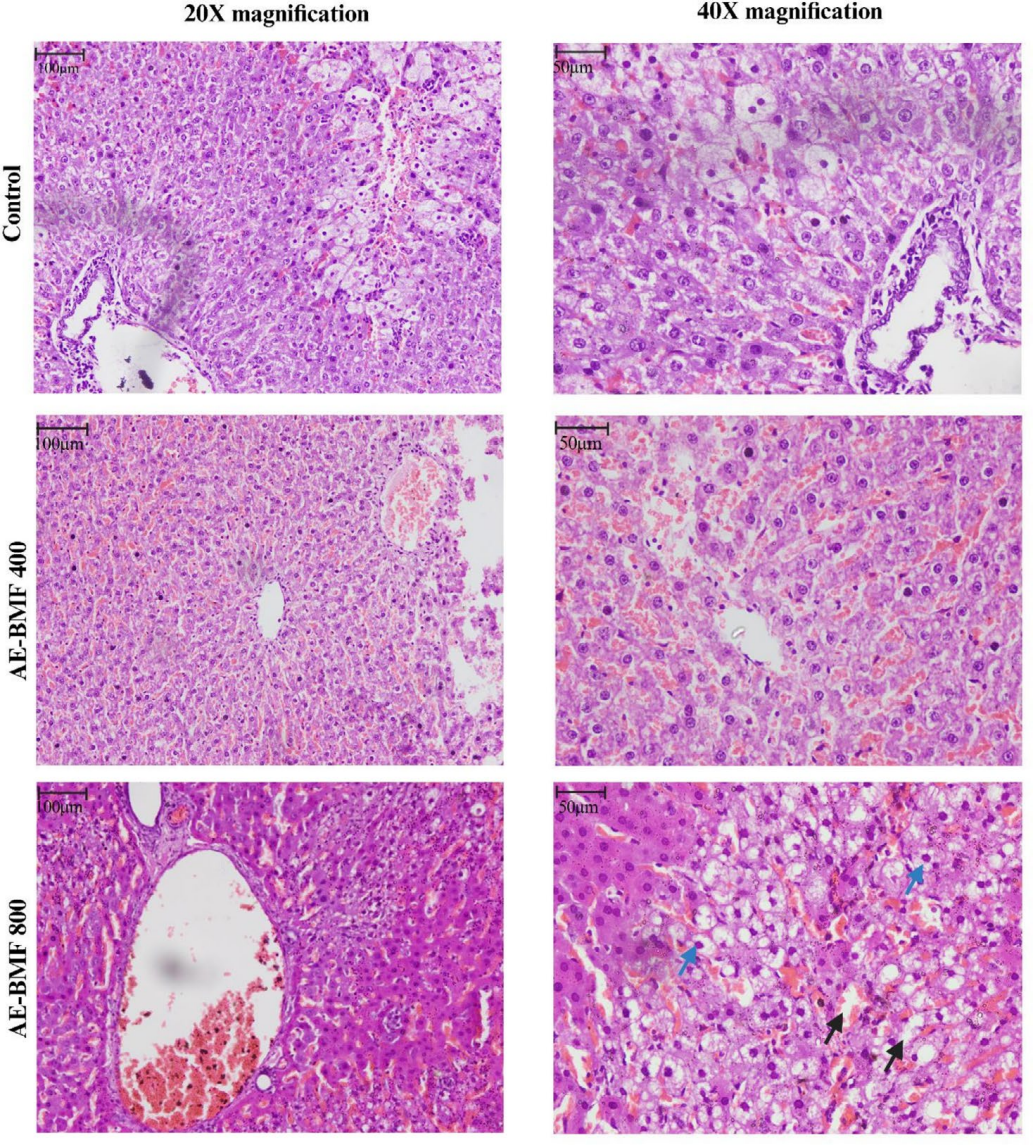
Effect of AE‐BMF on the liver sections of rats. The black arrow indicated hepatic fibrosis, and the blue arrow indicated inflammatory cell infiltration. AE‐BMF, aqueous extract of *B. motleyana* fruit.

**FIGURE 3:**
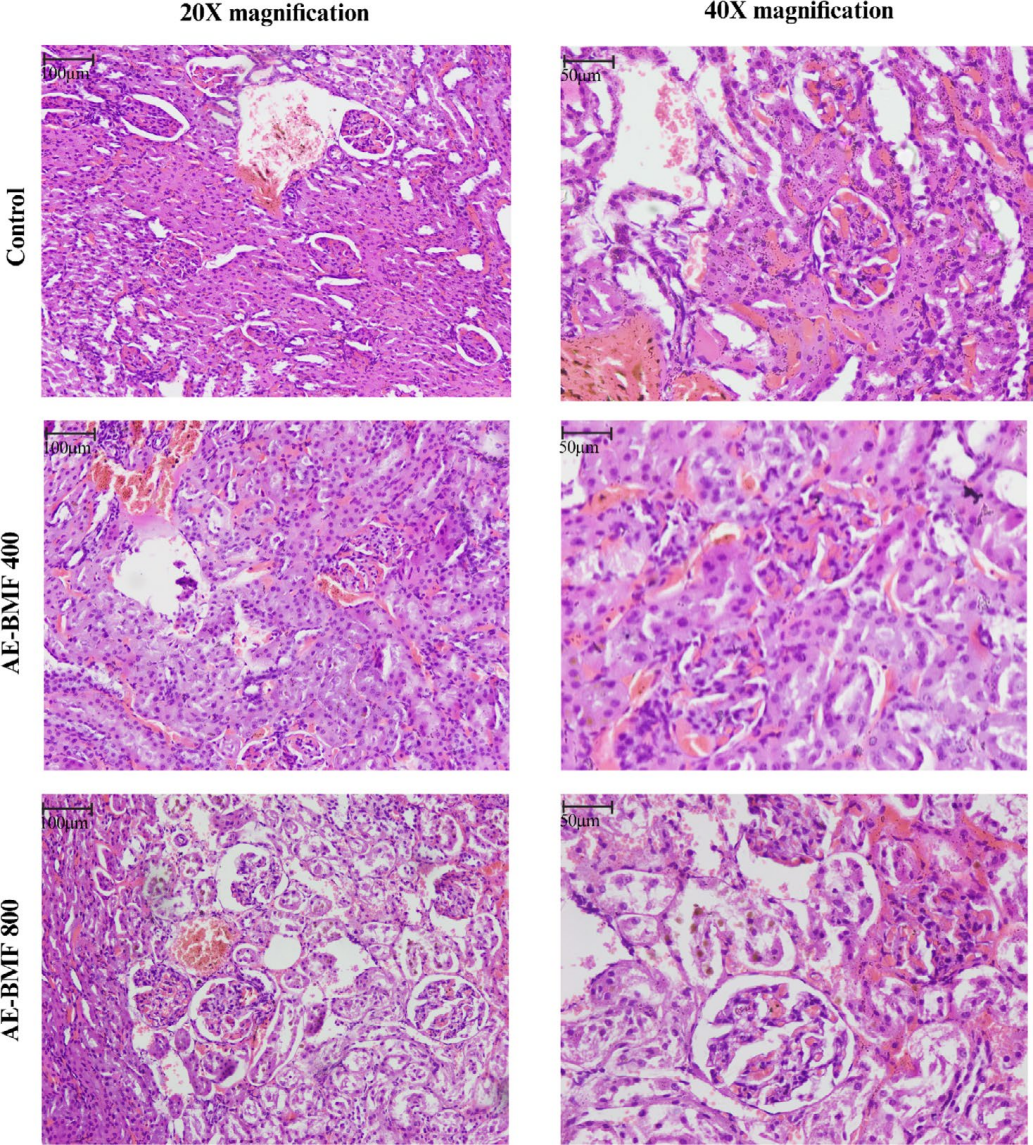
Effect of AE‐BMF on kidney sections of rats. AE‐BMF, aqueous extract of *B. motleyana* fruit.

Histological analysis revealed standard glomerular and tubular architecture in the kidneys of all groups. Liver sections from the control and AE‐BMF 400 mg/kg groups showed normal hepatocyte morphology and sinusoidal arrangement. In contrast, at 800 mg/kg, mild hepatic alterations were noted, including slight fibrosis, focal inflammatory cell infiltration, and minimal hepatocyte ballooning (Figures 2 and 3).

## 4 Discussion

The present research assessed the acute and subacute oral toxicity of AE‐BMF in Wistar rats Acute administration of a single oral dose of 4000 mg/kg did not induce mortality or apparent signs of toxicity during the 14 day observation period, suggesting that the LD_50_ of AE‐BMF is greater than 4000 mg/kg. According to the OECD and Globally Harmonized System (GHS) classification, substances with LD_50_ values above 2000 mg/kg are considered to have low acute toxicity (Islam et al. 2025). Thus, AE‐BMF may be considered relatively safe for oral administration. Similar findings have been reported for other edible tropical fruits, such as *Nephelium lappaceum* and *Lansium domesticum* , where extracts demonstrated LD_50_ values above 2000 mg/kg, highlighting the general safety of polyphenol‐rich fruit extracts when consumed in high doses (Abdallah et al. 2022; Oliveira et al. 2023). In the subacute study, daily administration of AE‐BMF for 28 days at 400 mg/kg and 800 mg/kg did not result in mortality or significant behavioral changes. However, animals treated with 800 mg/kg exhibited a significant reduction in body weight gain (*p* < 0.001), food intake (*p* < 0.001), and water intake (*p* < 0.05) compared with controls. In contrast, the 400 mg/kg group remained comparable to untreated animals. A decrease in body mass and food consumption has been previously described for fruits rich in tannins (Cosme et al. 2025a); (Li et al. 2022) and glycosides (Ulusoy et al. 2024), which are known to reduce palatability (Cosme et al. 2025b) and interfere with nutrient absorption (Fabbrini et al. 2022). For instance, a study reported a 19%–29% reduction in body weight in rats treated with high doses of *Cissus polyantha* aqueous extract, suggesting a shared mechanism involving phytochemicals that influence digestive efficiency (Miaffo, Kamgue, et al. 2025). Organ weight analysis revealed that AE‐BMF did not alter the weights of the kidneys, lungs, heart, spleen, or reproductive organs. Still, a significant increase in relative liver weight was observed at 800 mg/kg (+15.8%, *p* < 0.05). A modest elevation of liver mass is often interpreted as an adaptive response to metabolic stimulation, particularly in the presence of phytochemicals that induce hepatic enzymes (Prajapati et al. 2025). Consistent with this observation, serum ALT levels increased significantly in the 800 mg/kg group (+21.5%, *p* < 0.05), while AST remained unchanged. The selective rise in ALT without AST suggests mild hepatocellular adaptation rather than overt necrosis (Kalas et al. 2021a). Comparable results have been described in studies of *Combretum molle* and *Cissus polyantha*, where high extract doses elevated ALT while leaving AST unaffected, reflecting limited hepatic stress at pharmacological levels (Kalas et al. 2021b); (Miaffo, Doudou, et al. 2025a).

A more favorable biochemical outcome was observed, with a marked reduction in creatinine (−30.5%, *p* < 0.01) and uric acid (−28.9%, *p* < 0.01) at 800 mg/kg, indicating that AE‐BMF did not compromise renal function and may even enhance renal clearance (Miaffo, Doudou, et al. 2025a). These findings parallel those of Miaffo et al., who observed significant decreases in serum uric acid following treatment with *Cissus polyantha* stem extracts, attributed to polyphenolic compounds with uricosuric activity (Miaffo, Doudou, et al. 2025b). Moreover, AE‐BMF significantly decreased serum total cholesterol (−27.5%, *p* < 0.01) and triglycerides (−43.6%, *p* < 0.001) in the 800 mg/kg group. This hypolipidemic effect is consistent with the high phenolic and flavonoid content of *B. motleyana* , which has been previously reported to possess antioxidant and lipid‐lowering properties (Debnath et al. 2022b); (Liu et al. 2022); (Tu et al. 2022). Such reductions in circulating lipids are of therapeutic interest (Tokgözoğlu and Libby 2022), as elevated cholesterol and triglycerides are major risk factors for atherosclerosis and cardiovascular disease (Farnier et al. 2021). The lipid‐lowering activity observed in this study is in agreement with prior reports on other tropical fruits, including *Mangifera indica* and *Garcinia cambogia*, whose phenolic‐rich extracts produced comparable decreases in lipid parameters (Amini et al. 2024).

Hematological analysis demonstrated significant improvements in several parameters following AE‐BMF administration. RBC and hemoglobin levels increased at both 400 mg/kg and 800 mg/kg, with a highly significant effect at the higher dose, whereas hematocrit remained essentially unchanged. Platelet counts were markedly elevated in both treated groups, indicating enhanced thrombopoietic activity. Notably, lymphocyte percentages rose substantially at 800 mg/kg (+23.9%, *p* < 0.01), suggesting a strong immunostimulatory effect (Ciesielska‐Figlon et al. 2022). AE‐BMF treatment may be attributed to its phytochemical constituents, particularly flavonoids, phenolic compounds, and micronutrients, which are known to enhance hematopoiesis following the elevation of RBCs and platelets (Izuegbuna 2022); (Saha et al. 2025).

Erythropoietin production, which improves erythropoiesis and results in increased RBC counts and hemoglobin levels, can be stimulated by flavonoids (Liu et al. 2025). Phenolic compounds and natural antioxidants can reduce oxidative stress in bone marrow progenitor cells, thereby supporting normal RBC formation (Marcucci et al. 2023). Extracts rich in flavonoids and tannins are responsible for the increase in platelet counts, which may be linked to the ability of polyphenols to stimulate megakaryocyte proliferation and differentiation (Sharifi‐Rad et al. 2022). In plant‐derived preparations, such as *Moringa oleifera* and *Camellia sinensis* , similar hematopoietic effects have been documented, which enhanced both erythropoiesis and thrombopoiesis in experimental models (Prabowo et al. 2021). To enhance lymphocyte proliferation and immune surveillance, such an effect is consistent with the known ability of dietary flavonoids. For polyphenolic extracts of *Camellia sinensis* and *Moringa oleifera* , similar increases in lymphocyte counts have been described, further supporting the immunomodulatory potential of AE‐BMF (Prabowo et al. 2021).

Histopathological assessment confirmed the biochemical findings: the kidney architecture remained normal at all doses. In contrast, liver sections from the 800 mg/kg group revealed only mild inflammatory infiltration, mild fibrosis, and hepatocyte ballooning. Given the minimal alterations observed, there is no risk of severe hepatotoxicity (Li et al. 2023). Even at relatively high doses of AE‐BMF, the combination of limited histological changes, moderate ALT elevation, and preserved AST activity suggests no significant hepatotoxic risk in the short term (Zeng et al. 2022).

AE‐BMF is relatively safe, as indicated by an LD_50_ greater than 4000 mg/kg and by the absence of significant systemic toxicity at doses up to 800 mg/kg for 28 days. In addition to mild immunostimulatory effects, the extract exhibited beneficial hypolipidemic and uric acid‐lowering effects. These findings suggest additional potential pharmacological applications and support the traditional use of *B. motleyana* as a food source. Nonetheless, the slight hepatic alterations observed at high doses underscore the need for further subchronic and chronic studies, as well as mechanistic investigations to elucidate the pathways underlying its lipid‐lowering and immunomodulatory actions.

## 5 Limitation and Conclusion

Although it has several limitations, this study sheds light on the subacute toxicity of AE‐BMF. It was limited to 28 days; long‐term effects are unknown. Mild liver alterations and increased liver weight at 800 mg/kg suggest a possible dose‐related effect. Recovery groups were excluded; it is unclear whether these alterations are reversible or progressive. Other organ‐specific effects were not evaluated; only specified biochemical, hematological, and histological characteristics were analyzed. The study employed healthy rats; outcomes may differ in illness models or humans. To determine whether the observed effects are reversible, future studies should incorporate a recovery phase. Additionally, the inclusion of satellite groups would be beneficial for a more accurate assessment of the reversibility or irreversibility of any potential adverse effects.

In conclusion, AE‐BMF appears to be relatively safe at low to moderate doses, showing no mortality, major behavioral changes, or significant organ toxicity at 400 mg/kg. At higher doses (800 mg/kg), AE‐BMF induced mild reductions in body weight, food and water intake, moderate alterations in liver function, and minor histological changes in the liver, indicating a threshold for potential toxicity. Overall, AE‐BMF exhibits a favorable safety profile at doses below 800 mg/kg, supporting further exploration of its pharmacological applications; however, caution is warranted at higher doses.

## Author Contributions

**Mohammed Kamrul Hossain:** methodology, software, supervision, project administration, resources, writing – review and editing. **Eftekhar Alam Bhuyan:** methodology, software, writing – review and editing. **Md. Mahmudul Hasan:** conceptualization, writing – review and editing, writing – original draft, methodology, software, visualization. **Nawreen Monir Proma:** methodology, project administration, supervision, software, data curation, writing – review and editing.

## Funding

The authors have nothing to report.

## Ethics Statement

The Ethical Review Board approved this study. Departmental ethical consent number AERB‐FBSCU‐20250615‐(1).

## Conflicts of Interest

The authors declare no conflicts of interest.

## Data Availability

The data that support the findings of this study are available from the corresponding author upon reasonable request.
